# A spike is a spike: On the universality of spike features in four epilepsy models

**DOI:** 10.1002/epi4.13062

**Published:** 2024-10-09

**Authors:** Armen Sargsyan, Pablo M. Casillas‐Espinosa, Dmitri Melkonian, Terence J. O'Brien, Gilles van Luijtelaar

**Affiliations:** ^1^ Kaoskey Pty Ltd Sydney New South Wales Australia; ^2^ Orbeli Institute of Physiology Yerevan Armenia; ^3^ Department of Medicine, the Royal Melbourne Hospital The University of Melbourne Parkville Victoria Australia; ^4^ Department of Neuroscience, Central Clinical School Monash University Melbourne Victoria Australia; ^5^ Department of Neurology The Alfred Hospital Melbourne Victoria Australia; ^6^ Donders Centre for Cognition Radboud University Nijmegen the Netherlands

**Keywords:** EEG frequency analyses, genetic absence models, post‐SE model, post‐traumatic epilepsy model, spike–wave discharges, temporal lobe epilepsy

## Abstract

**Objective:**

Frequency properties of the EEG characteristics of different seizure types including absence seizures have been described for various rodent models of epilepsy. However, little attention has been paid to the frequency properties of individual spike–wave complexes (SWCs), the constituting elements characterizing the different generalized seizure types. Knowledge of their properties is not only important for understanding the mechanisms underlying seizure generation but also for the identification of epileptiform activity in various seizure types. Here, we compared the frequency properties of SWCs in different epilepsy models.

**Methods:**

A software package was designed and used for the extraction and frequency analysis of SWCs from long‐term EEG of four spontaneously seizing, chronic epilepsy models: a post‐status epilepticus model of temporal lobe epilepsy, a lateral fluid percussion injury model of post‐traumatic epilepsy, and two genetic models of absence epilepsy—GAERS and rats of the WAG/Rij strain. The SWCs within the generalized seizures were separated into fast (three‐phasic spike) and slow (mostly containing the wave) components. Eight animals from each model were used (32 recordings, 104 510 SWCs in total). A limitation of our study is that the recordings were hardware‐filtered (high‐pass), which could affect the frequency composition of the EEG.

**Results:**

We found that the three‐phasic spike component was similar in all animal models both in time and frequency domains, their amplitude spectra showed a single expressed peak at 18–20 Hz. The slow component showed a much larger variability across the rat models.

**Significance:**

Despite differences in the morphology of the epileptiform activity in different models, the frequency composition of the spike component of single SWCs is identical and does not depend on the particular epilepsy model. This fact may be used for the development of universal algorithms for seizure detection applicable to different rat models of epilepsy.

**Plain Language Summary:**

There is a large variety between people with epilepsy regarding the clinical manifestations and the electroencephalographic (EEG) phenomena accompanying the epileptic seizures. Here, we show that one of the EEG signs of epilepsy, an epileptic spike, is universal, since it has the same shape and frequency characteristics in different animal models of generalized epilepsies, despite differences in recording sites and location.

**Key points**

- The spectral properties of the epileptic spike—the fast component of the spike–wave complexes constituting epileptiform activity accompanying various seizure types, are identical in four different rat models of epilepsy.
- The amplitude spectrum of the epileptic spike has a single peak at frequency close to 20 Hz in all models.
- The similarity of spike properties in different epilepsy models suggests that a common mechanism may be responsible for spike generation.
- There was noticeable cross‐model diversity in the frequency of the slow component (wave) of the spike–wave complexes.

## 1 INTRODUCTION

Epilepsy is characterized by recurrent and sudden incidence of epileptic seizures, which are the result of excessive neuronal discharges in the electroencephalogram (EEG)—the representative of the global electrical activity of the brain.^1^ The most characteristic feature of EEG seizures is the occurrence of repetitive spikes or polyspikes that are the reflection of synchronized firing of large number of neurons and often followed by slow waves, representing neuronal silence.^2^

Animal models play an essential role in investigating and understanding the biological mechanisms and pathophysiological processes underlying the different epilepsy types. For the study of acquired epilepsy, we used two most commonly used models: the kainic acid (KA) induced post‐status epilepticus (post‐SE),^3^, ^4^, ^5^, ^6^, ^7^ and the post‐traumatic brain injury (PTE) models.^8^, ^9^, ^10^, ^11^ The Genetic Absence Epilepsy rats from Strasbourg (GAERS) and Wistar Albino Glaxo/from Rijswijk (WAG/Rij) were used as models for genetic generalized absence epilepsy.^12^, ^13^, ^14^, ^15^, ^16^

Despite significant differences in seizure biology, seizure duration and intensity, and temporal dynamics between these models, the EEG contains certain common elements. To illustrate, the seizures described in all four models are accompanied by regularly repeating and pronounced trains of spike and waves in the EEG—a widespread ictal pattern presumably generated by synchronous and periodic activation and deactivation of a large number of cortical neurons reciprocally interconnected with the thalamus or other regions.

A spike–wave complex (SWC), the fundamental EEG element in spike‐and‐wave trains, consists of two prominent components: a fast and a slow component. The fast component consists of a spike and a preceding and following transient with polarities opposite to that of the spike. The slow component shares the spike's polarity and forms the slow wave that follows the initial three transients.^17^, ^18^ The wave is strongly associated and coupled to the preceding spike and they necessarily follow each other in time, as demonstrated in numerous clinical and experimental studies.^19^, ^20^, ^21^

The frequency properties of epileptiform activity in the EEG including trains of SWCs (i.e. spike–wave discharges [SWDs]) have been thoroughly investigated in various genetic and induced rodent models of epilepsy.^18^, ^22^, ^23^, ^24^, ^25^, ^26^ However, little attention was paid to the frequency properties of individual SWCs, the constituting element of EEG seizures across different models of genetic and acquired epilepsies.

Understanding SWC's properties may be crucial for gaining better insights into the dynamic characteristics of the underlying systems that generate this specific component of ictal activity. Moreover, this knowledge can be applied to automated seizure detection methods.^6^ It is important to know whether the properties of SWCs, and, particularly, their frequency characteristics, have commonalities or dissimilarities for the different seizure types. Previously, we observed similarities in the spectral properties of SWCs across different rat models, but no quantitative analysis was conducted to confirm the consistency and reliability of this observation.^6^

Here, we performed a comparative analysis of the frequency properties of individual spikes and waves of SWCs in the generalized seizures across four spontaneously seizing chronic epilepsy rat models: two acquired and two genetic generalized models.^27^

## 2 METHODS

### 2.1 Terminology of key concepts in this paper

To avoid confusion, we define the terms used in this article as follows:

Seizure—denotes the clinical phenomena in the acquired and genetic absence models and is accompanied by epileptiform activity in the EEG (Figure 3A).

Spike–wave discharge (SWD)—a train of recurring SWCs, lasting minimally 1 s.

Spike–wave complex (SWC)—one cycle of the recurring EEG activity during a seizure (Figure S1, Figure 3B, gray traces) as defined by Weir^17^ and Sitnikova and van Luijtelaar.^18^

Spike complex (SC)—the fast component of the SWC consisting of spike and preceding and following transients of opposite polarity that remains after removing the slow wave (Figure 3B, black traces).

### 2.2 Animals

Eleven‐week‐old male Wistar rats served as experimental subjects for the post‐SE and PTE models, eight animals for each model. Eight 24‐week‐old male GAERS and eight male WAG/Rij rats, age >6 months represented the genetic epilepsy models. All procedures were approved by the ethics committees, in Australia by the Florey Animal Ethics Committee (ethics number 14‐072 UM for the post‐SE, PTE and GAERS models) and by the DEC, Radboud University, Nijmegen, the Netherlands for the WAG/Rij model. Animals in both labs were individually housed in a temperature‐controlled vivarium with alternating 12‐h light and dark cycles. Food and water were provided ad libitum throughout the whole study. The post‐SE and PTE models were generated as previously described^5^, ^6^, ^8^, ^10^, ^27^, ^28^, ^29^, ^30^ and the methodologies are expanded in Supporting Information 1.

### 2.3 ECoG electrode implantation and EEG acquisition

ECoG surgery was performed in both labs under isoflurane anesthesia: 10 weeks after SE in the post‐SE model, 6 months post‐fluid percussion injury in the PTE model, at 6 months of age for the GAERS, and between 6 and 8 months for the WAG/Rij. All procedures were previously described^27^ and are detailed in Supporting Information 1.

### 2.4 EEG recordings

EEG for the GAERS, post‐SE and PTE models were acquired using Profusion 5 software (Compumedics, Australia) unfiltered and digitized at 512 Hz, at Radboud, a WINDAQ data acquisition was used. Rats in both laboratories were allowed to freely move during the recordings in their home cages. Referential montages were used in all four models with reference electrode located over the cerebellum and active electrode over frontal (WAG/Rij) or frontoparietal (GAERS, post‐SE and PTE) cortex. The signals were band pass filtered (1 and 100 Hz).^27^, ^31^ EEG recordings were acquired for 2–4 weeks continuously in the post‐SE and PTE rats whilst GAERS and WAG/Rij had 48 h of continuous EEG recordings.^16^ All GAERS and WAG/Rij rats had spontaneous occurring SWDs lasting between 1 and 20 s; regarding the post‐SE and FPI models: only animals that presented with epileptiform activity in the EEG were included.

### 2.5 EEG analysis

EEG analysis was performed using the Kaoskey (Sydney, Australia) software package. This in Delphi (Embarcadero Technologies) written package, contains several modules allowing for manual and automatic selection of specific EEG or ECoG fragments and calculation of their Fourier transform. The software contains also modules for signal preprocessing and averaging of the selected fragments.

**FIGURE 1:**
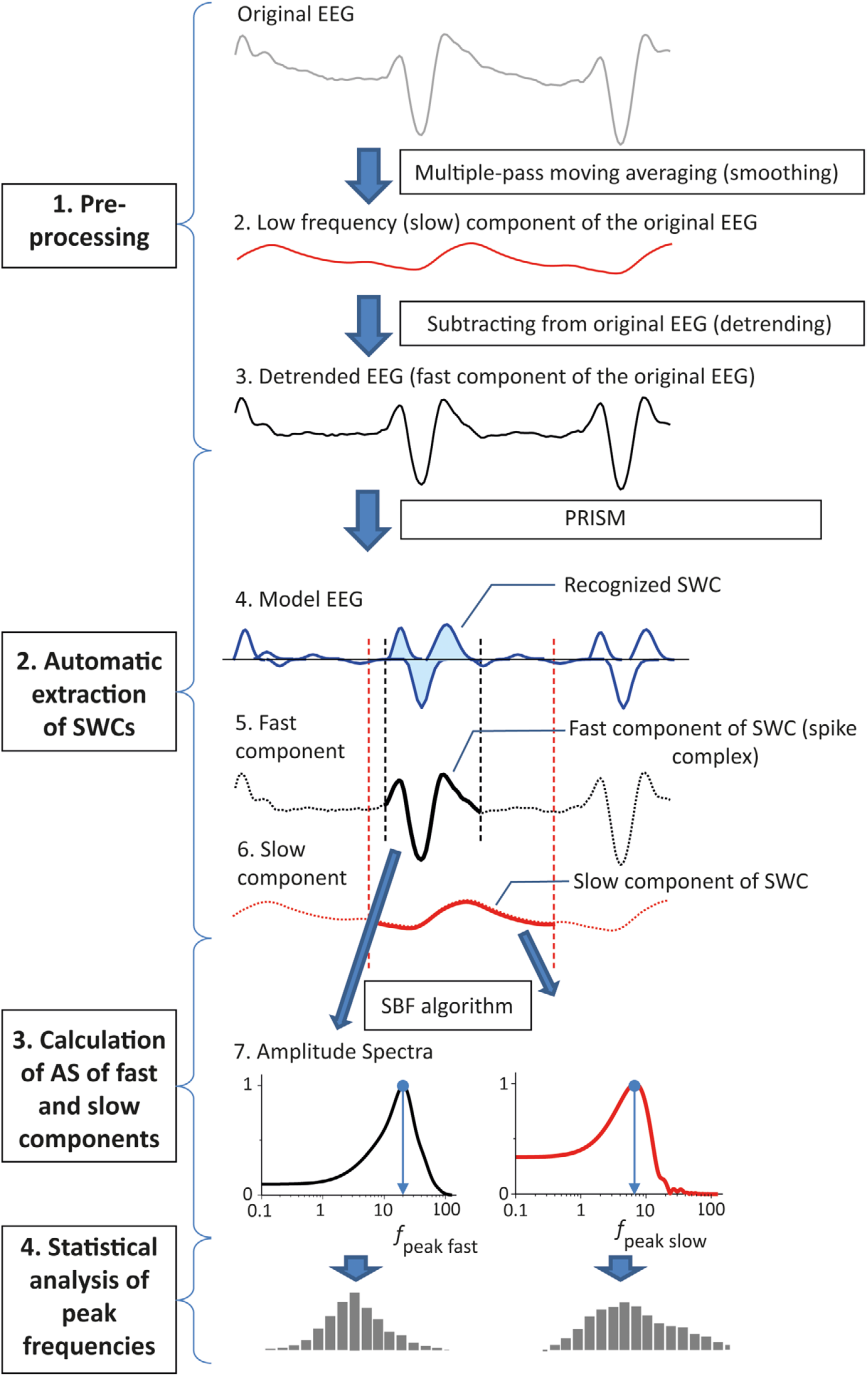
Flow chart illustrating EEG processing steps.

The analysis of the EEG signal was divided into four steps (Figure 1).

#### 2.5.1 Preprocessing

Preprocessing separated the original EEG (Figure 1, trace 1) into two components: a low‐frequency component, which included the baseline trend and the slow waves present in the epileptiform activity (Figure 1, trace 2), and a fast component, which included the spikes or spike complexes (Figure 1, trace 3). The low frequency component was calculated using a two‐pass two‐sided moving average^32^ (40 ms window, 20 ms on each side of the central point), next, this was subtracted from the original EEG—a process that we refer to as detrending. Examples of detrended SWC are presented in Figure 3B.

#### 2.5.2 Automatic extraction of SWC

The automatic extraction of SWCs was based on the proprietary algorithm of pattern recognition that consists of two major parts: 1) creating a model of the input EEG by applying the fragmentary decomposition (FD) algorithm^33^ and 2) amodel‐based detection of specific patterns.

The FD algorithm^33^ transforms the input signal into a linear aggregation of multiple sub‐components—a sequence of quasi‐Gaussian kernels (QGK) fitted to the half‐waves of the input signal. Each QGK is fully defined by three parameters: amplitude, shape, and onset time. The algebraic sum of these QGKs forms the model signal that accurately resembles the input signal (Figure 1, trace 4). The second part of the algorithm analyses the model EEG and finds the sequences of QGKs that match a specific template pattern. The template is also a sequence of QGKs that describes the typical morphology of an SWC.

The algorithm was applied to the fast component of the EEG to detect the SCs. The preparation of the template pattern for SC detection was done manually for each animal/channel. Template patterns consisted of three QGKs, corresponding to the three‐phasic spike components PT_early_, Sp_2_, and PT_late_.^18^ The software then ran automatically through the entire EEG and detected the fragments that matched the template. Detected SCs that had no following or preceding SCs within an interval of 0.5 s were considered as interictal spikes and were excluded from further analysis.

**FIGURE 2:**
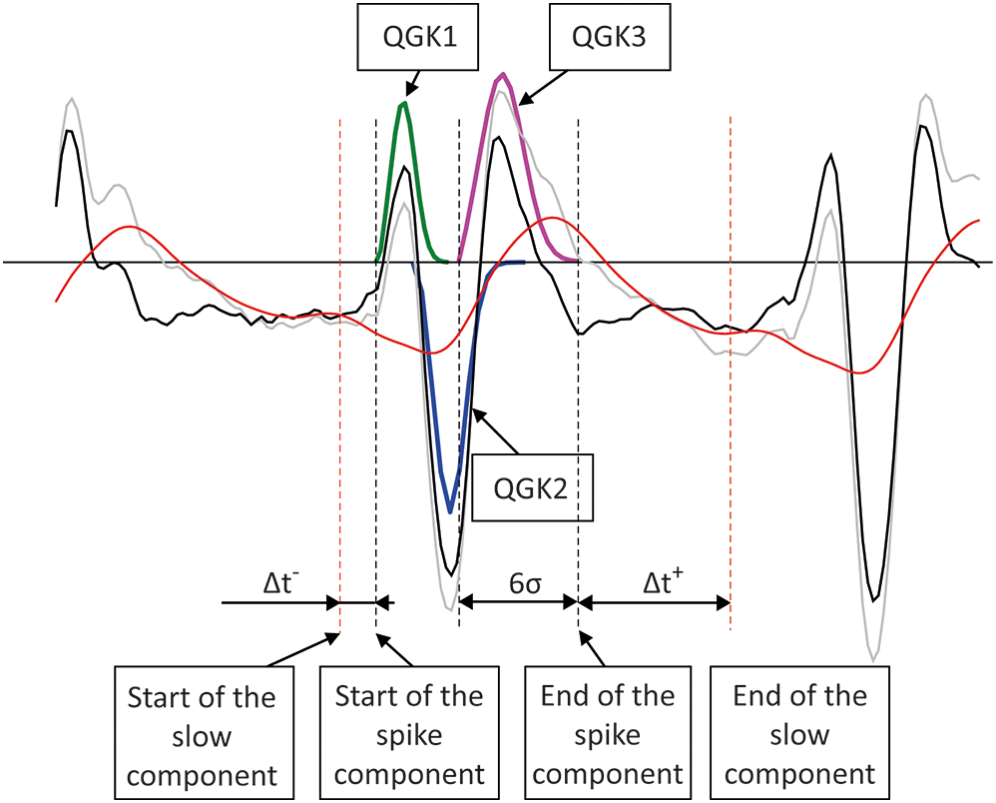
Illustration of automatic extraction of the fast (SC) and slow components of the SWC. Gray trace: The original EEG; red trace: The low frequency component obtained after applying multiple‐pass moving average to the original EEG; black trace: The difference between the original EEG and the low frequency component. QGK1, QGK2, QGK3—the elements of the signal model of a single SWC obtained after the application of fragmentary decomposition to the fast component. Start of the fast (spike) complex is taken as the onset time of the first element (QGK1) of the model. End of the SC is taken as the onset time of the third element of the model (QGK3) plus 6 times σ (the shape parameter) of the third element. The start of the slow component is taken as the start of the SC minus some fixed time interval, Δt^−^; the end of the slow component is taken as the end of the SC plus some other fixed time interval, Δt^+^. The time intervals Δt^−^ and Δt^+^ were defined manually for every record so that the entire interval of the slow component of the SWC does not overlap with the preceding and following SWCs.

The start and end times of each detected SC complex were defined as described in Figure 2, and the corresponding fragment was extracted from the detrended EEG (Figure 1, trace 5). The corresponding slow component was extracted from the low frequency component obtained at the EEG preprocessing stage (Figure 1, trace 6). Since the slow component is longer than the SC, its end time was defined by extending the end time defined for the SC by a fixed time interval. This added time interval was defined for each recording before the automatic processing so that it approximately covered the inter‐spike interval, but did not overlap with the next spike (Figure 2). The fast and slow components of the detected segments were further processed separately (Figure 1, traces 5, 6).

#### 2.5.3 Calculation of amplitude spectra (AS) of fast and slow components

For each extracted SC and its corresponding slow component, the AS (Figure 1, trace 7) was calculated using the similar basis function (SBF) algorithm for Fourier Transform.^34^ The averaged SC's and slow component's time‐courses and their AS were also calculated for each animal. A detailed description of AS calculation is given in Supporting Information 1.

#### 2.5.4 Statistical analyses of the peak frequencies

The peak value of AS of all detected SC, as well as its average, mode and standard deviation are given in Table S4. A similar analysis was performed for the slow component (Table S5). Kolmogorov–Smirnov and Shapiro–Wilk tests showed that the AS's peak frequencies were normally distributed. Therefore, the differences between the four epilepsy models were tested with a parametric ANOVA with epilepsy models as “between subjects” factor, followed by Bonferroni's post‐hoc tests, if appropriate.

It should be noted that since SWCs and SCs from different animals or even from different channels of the same animal may show differences in morphology (due to, e.g., different electrode locations or different positions of the reference electrode) the above analysis, especially the averaging, was performed separately for each channel and each animal.

## 3 RESULTS

### 3.1 SWC mimic each other

**FIGURE 3:**
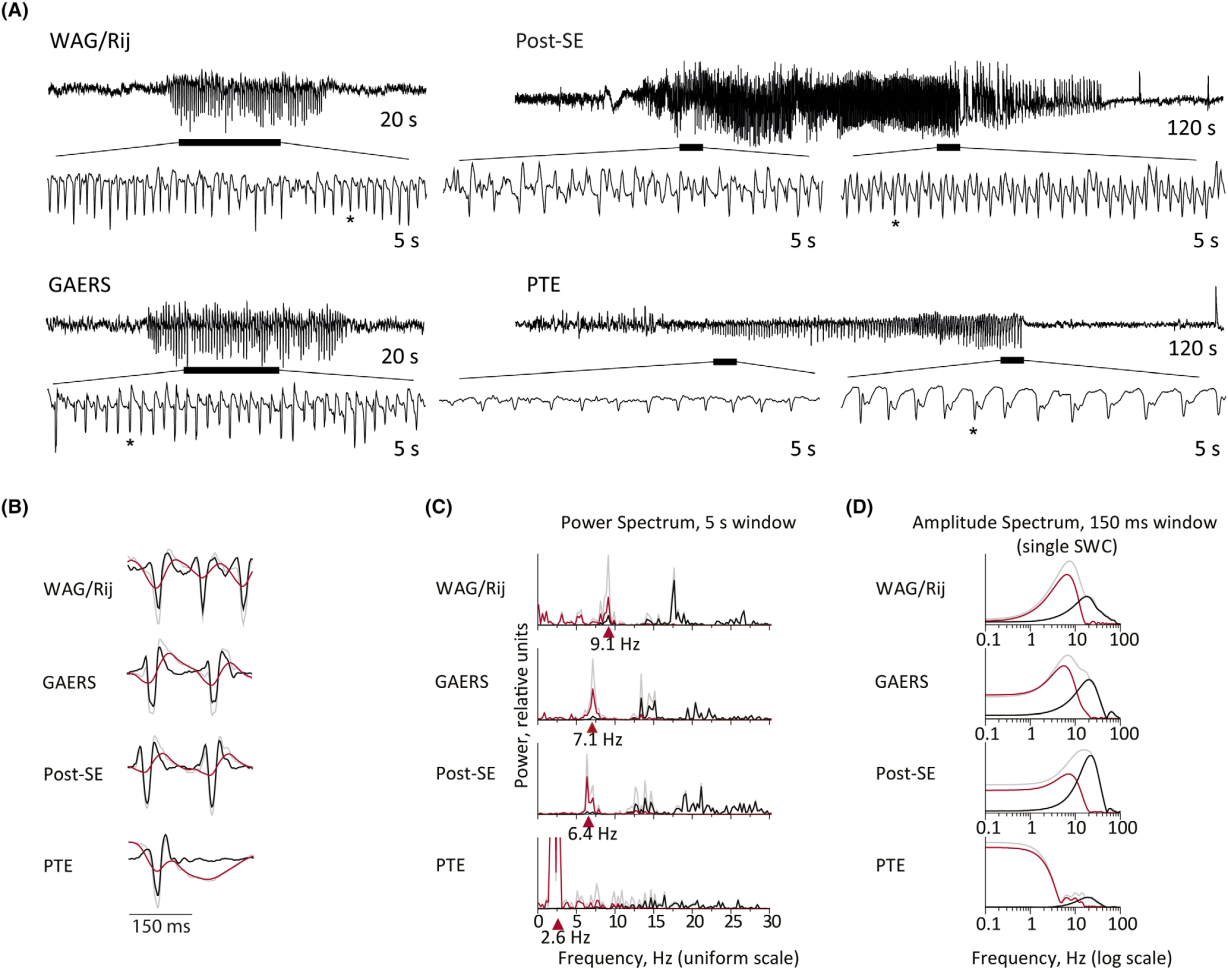
(A) Examples of representative types of epileptiform EEG activities from the four rat models. Two (for WAG/Rij and GAERS) or three (for post‐SE and PTE) traces are shown; the upper trace shows the entire seizure (20 s EEG fragments for WAG/Rij and GAERS, 120 s EEG fragments for post‐SE and PTE); the lower traces show enlarged 5 s fragments from the upper trace. The horizontal bars below the upper traces show the position of the corresponding 5 s fragment. The two 5 s fragments for post‐SE or PTE seizure are selected from the initial and the final epileptiform parts of the EEG, correspondingly, to show the pattern variations. (B) Short (300 ms) fragments of traces in A (the first SWC in the short fragment is marked by an asterisk in the corresponding trace in A). The slow component of the EEG (red line) was subtracted from the original EEG (gray line) to obtain the preprocessed EEG (with the fast, or spike, component, black line). (C) Power spectra of the 5 s EEG fragments shown in A (traces with “*”). Gray line: Power spectrum of the original EEG; red line: Power spectrum of the slow component removed during preprocessing; black line: Power spectrum of the preprocessed EEG. Note that the power spectrum peak for original EEG of the PTE model is truncated (the vertical scale is adjusted to show the higher frequency peaks). Red triangles indicate the largest peak of the power spectrum of the original EEG, which in all models coincides with the largest peak of the slow component. This peak's frequency is close to the repetition rate of SW complexes, or mean discharge frequency, within the corresponding 5 s EEG fragment calculated by dividing the number of SWCs in the fragment by the duration of the fragment (8.8 Hz for WAG/Rij, 7 Hz for GAERS, 6.4 Hz for post‐SE, and 2.4 Hz for PTE). Note that these frequencies are significantly different for some of the different models. These peaks are also significantly reduced by the preprocessing. (D) Effect of preprocessing on the AS of a single SW complex. The AS are calculated for the corresponding 150 ms EEG fragments containing a single SW complex shown in (B) Gray line—AS of original SW complex, red line—AS of the slow component, black line—AS of preprocessed SW complex.

The examples of epileptiform EEG activity in the four models are shown in Figure 3A and Figure S2. Note that the waveforms of individual SWCs from different rat models have a similar structure and temporal dynamics, especially after preprocessing (Figure 3B), despite remarkable differences in the mean discharge frequency (Figure 3A,C).

### 3.2 The effect of preprocessing

The detrending, as expected, had a large impact on the frequency characteristics of the epileptiform EEG activity, especially at the lower frequencies (Figure 3C,D). Note that the largest peaks of the power spectra of the original EEGs of the four models were all at frequencies below 10 Hz (Figure 3C). These low frequencies represent the mean frequency of the epileptic activity of the four models and coincide with the frequency of occurrence of the SWCs in a train of SWCs. The peaks of the power spectra of the low frequency component practically coincide with the peaks of the power spectra of the original EEG.

The ASs of the SCs of different models after detrending became quite similar and peak at about the same frequency (~20 Hz), whereas the ASs of the original SWCs and the slow component have high values and peaks at lower (0.1–10 Hz) frequencies (Figure 3D).

### 3.3 Automated detection of SWC in the four epilepsy models

To reveal whether there are inter‐model differences or similarities of frequency properties of the fast component of SCs as part of the generalized SWDs, an automatic procedure of spike–wave detection was applied to recordings from eight different animals from each epilepsy model; 32 recordings in total, record durations from 2 h to 12 days. The parameters of the procedure were defined for each recording to include the majority (>90%) of the SCs. A visual inspection of all seizures in post‐SE and PTE models and of 15–20 SWDs per record for the WAG/Rij and GAERS ensured that most of the SCs within the generalized SWDs were correctly selected and included. The late phase SCs in the post‐SE and PTE were always correctly detected. Interictal spikes (<4% on average) were automatically excluded by the algorithm. In total, 104 510 SCs were identified, processed, and statistically analyzed.

There were also improper detected events (<5%), their peak frequency was outside the 10–30 Hz range. Visual inspection of the EEG of these events showed that they were artifacts, and they were eliminated from further analysis.

### 3.4 Frequency analysis of the spike component

For every animal, the ASs of all identified SCs were calculated and the peak frequencies were determined. Figure S3 shows three arbitrarily selected SCs for each rat model and their corresponding ASs. Note that in all these examples the AS peaks close to 20 Hz.

**FIGURE 4:**
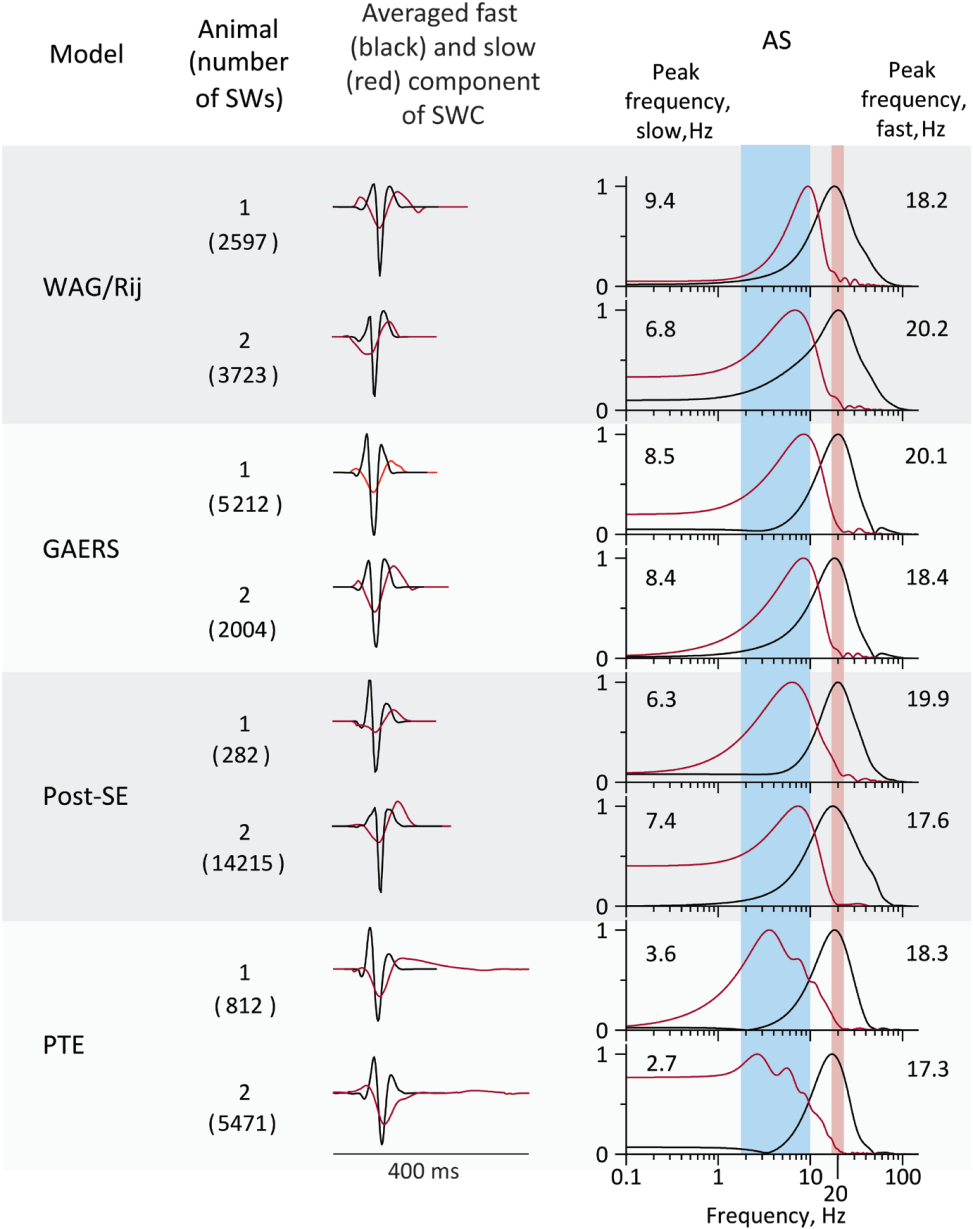
Averaged fast (left column, black traces) and slow (left column, red traces) components of the SWCs and their AS (AS, right column, black lines—AS of averaged fast component, red lines—AS of averaged slow component) for four different rat models of epilepsy. The averaged fast and slow components of the SWCs were calculated from EEG recordings from two different animals from each model. The number in parentheses shows the number of individual SWCs detected and averaged in each recording. The averaging of fast and slow components was performed separately. The durations of recordings were: WAG/Rij: 2 h (animals 1 and 2), GAERS: 23 h (animals 1 and 2), post‐SE: 26 h (animal 1) and 283 h (animal 2), PTE: 73 h (animals 1 and 2). The peak frequencies, in Hz, of the AS of fast and slow components are shown in the right column. The pink and blue bars in the right column show the scatter of the peak frequencies of AS of averaged fast and slow components, correspondingly, across 24 animals (4 models, 8 animals each).

**FIGURE 5:**
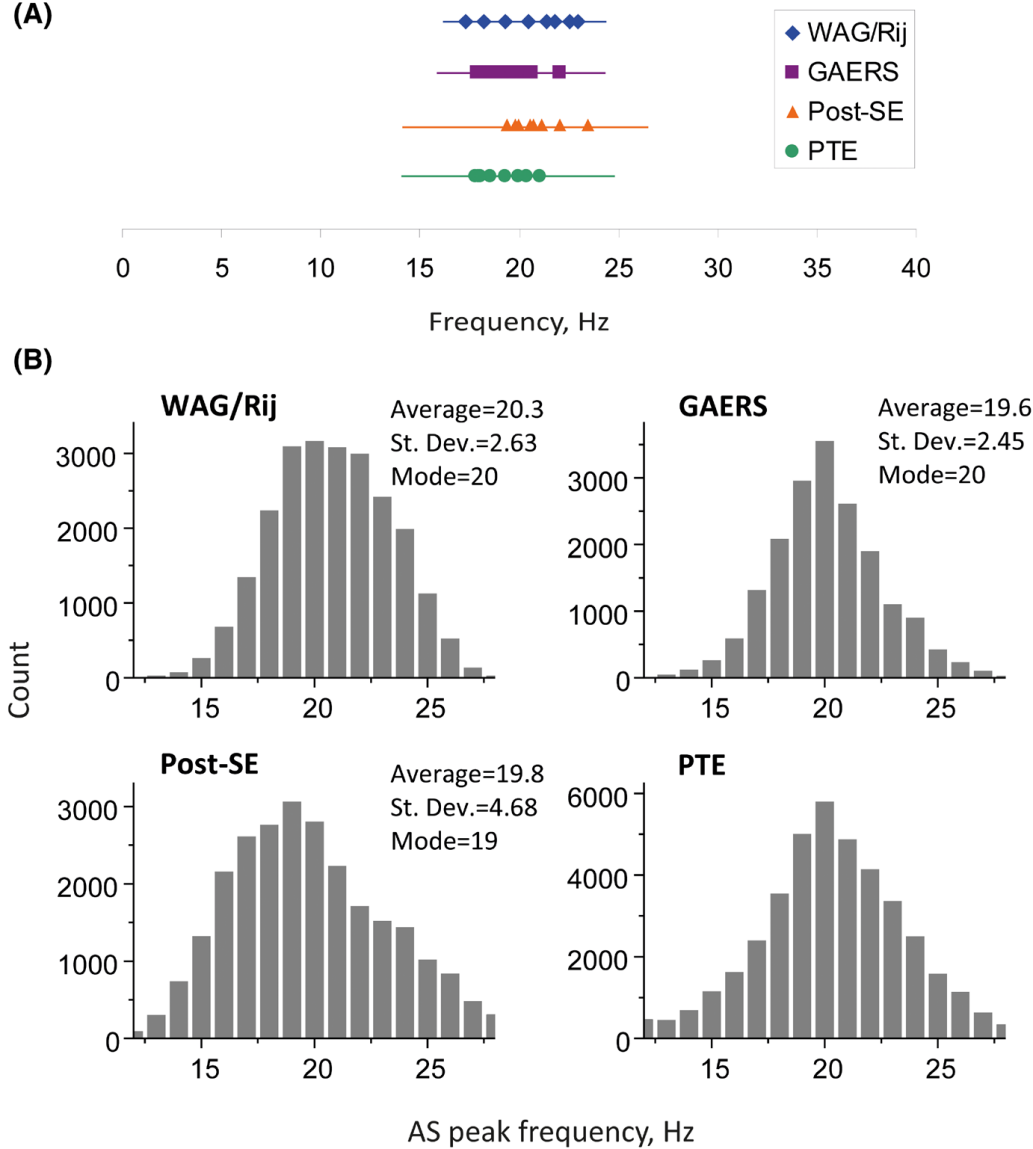
(A) Comparison of AS peak frequency of individual SCs across animals and models. The shapes (diamond, square, triangle, and filled circle) show the positions of the average AS peak frequency for each animal of WAG/Rij, GAERS, post‐SE and PTE models, correspondingly. The horizontal lines indicate ±1 standard deviation (columns 4 and 5 in Table S4). (B) Meta‐histograms of SC AS peak frequency distribution. Each histogram includes data (SC AS peak frequencies) from 8 different animals of the same model.

The mean, standard deviation and modes of the distribution histograms of AS peak frequencies for all correctly identified SCs per animal are presented in Table S4, and Figure 5A. The averaged SC waveforms and their ASs are depicted in Figure 4, their AS peak frequencies in Table S4. It follows from the data presented in Table S4 that individual SCs taken from the different models have a similar frequency composition with a single prevailing frequency close to 18–20 Hz.

We constructed histograms of peak frequency distributions by pooling the AS peak frequencies (Figure 5B) of individual SC to allow comparisons of AS peak frequencies across the four models. These histograms show that the SC AS peak frequency does not differ significantly between the models, confirming that this feature does not depend on the particular epilepsy model. This was confirmed by the outcomes of a one‐way ANOVA; it did not show significant F values for the peak frequencies of the AS between the models (*F* = 2.15, df 3.28, *p* = 0.12, *ή* = 0.19), and this was found for the mode (*F* = 0.46, df 3.28, *p* = 0.71, *ή* = 0.05) and averaged peak frequencies as well (*F* = 1.76, df 3.28, *p* = 0.18, *ή* = 0.16). Therefore, no significant differences were found for the average frequency and the histogram mode frequency at ca. 20 Hz for all models.

### 3.5 Frequency analysis of the slow components

In contrast to the AS of the SC, the ASs of the slow components showed larger variability within animals and epilepsy models. The average value of the AS peak frequencies of the slow component across 8 animals was 8.5 Hz for WAG/Rij, 8 Hz for GAERS, 4.5 Hz for post‐SE, and 4.5 Hz for PTE models. The results are presented in Table S5. The peak frequencies of the individual slow components were close to the main frequency of epileptiform activity (reciprocal of the inter‐spike interval) for a particular model. The distribution of the peak frequencies did not deviate from a normal distribution and an ANOVA could be used.

The analyses of the AS of the slow wave revealed between group effects for the peak frequency (*F* = 17.48, df 3.28, *p* < 0.001, *ή* = .65), the mode (*F* = 14.07, df 3.28, *p* < 0.001, *ή* = 0.60) and the peak averaged (*F* = 10.15, df 3.28, *p* < 0.001, *ή* = 0.52). The non‐parametric statistic of the mode showed the same group differences as the parametric analyses. The post hoc tests showed that the AS spectra of the genetic absence models did not differ from each other, also the post‐SE and PTE models did not differ from each other, but the two genetic models differed from the two acquired models: the genetic models had higher peak frequencies of the slow component compared to the induced models.

## 4 DISCUSSION

The main outcome of our comparative frequency analysis of epileptiform activity accompanying seizures in two acquired and two genetic models is the close resemblance of the spectral properties of the fast component of the SWC—the epileptic spike. The SWC consists of a sequence of a positive deflection, a large “negative” spike, and another “positive” deflection.^17^, ^18^ This fast component represents the potentials generated during the simultaneous activation of a large number of cortical pyramidal cells in different cortical layers in response to cortical or thalamic stimulation and includes potentials generated by excitatory and inhibitory synapses and by the transmembrane currents of pyramidal cells during the generation of action potentials. Most of these neurons are silent during the wave, as has been abundantly shown by multiple unit and intracellular recordings in different seizure and epilepsy models.^19^, ^20^, ^21^, ^35^, ^36^, ^37^

Removal of the slow component by preprocessing revealed that the SCs from the four models became similar in the time domain. This is reflected in the frequency domain as a remarkable similarity of the AS of a large number of single SCs from all four models, with a single prevailing frequency of 18–20 Hz. The similarity of different descriptive statistics of the SCs, including the average, histogram, and the peak frequency of averaged SC across all rat models, confirms the generality of our findings. This resemblance is striking, considering that the neurobiology and phenotypic characteristics of the different epilepsy models are rather different, including the morphology of the epileptic EEG activity, temporal and spatial dynamics, seizure duration, and frequency of occurrence. Furthermore, the similarities of the spikes across the models also withstand the methodological difference during EEG acquisition, including the montage, the location of the epidural recording, and reference and ground electrodes, factors known to modulate the morphology of the EEG signal. However, despite these differences in EEG acquisition methods, a spike in a train of cortical recorded SWDs is similar across the different epilepsy models and independent of the seizure type. It can be argued that the high pass filtering can result in false oscillations^38^ and that could be a reason for the similarities of the spike characteristics, however, the strong neurophysiological underpinning of SWCs and the outcomes of modeling studies make this possibility not very feasible.^19^, ^26^, ^35^, ^37^ The commonalities across the animal models may point toward similar underlying electrophysiological processes and spike constituent factors. An earlier computational modeling study showed that currents flowing through the membranes of cortical pyramidal neurons, the major contributors to cortical field potentials account for the three‐phasic spike part of the experimental found SWC (i.e. fast component).^37^ This mechanism could be common for different epilepsy models, especially when seizures become fully generalized and involve cortico‐cortical networks. However, the fact that the properties of the spike are similar does not prove that the mechanisms of spike generation are similar, too—spikes could still be generated by variable and diverse mechanisms.

Despite the fact that both GAERS and WAG/Rij are absence models of a cortico‐thalamo‐cortical network type of epilepsy and share many neurobiological similarities, such as the age‐dependent increase in SWDs, their pharmacological profile, a SWD initiating site in the somatosensory cortex and the crucial involvement of the reciprocal connected thalamus, the strains differ in their polygenic alterations presumed to cause the epileptic phenotype.^15^ There are differences in gene mutations,^14^, ^39^ phenomenological differences in the SWDs expression^23^ and in the age of onset.^24^, ^39^, ^40^, ^41^ However, in our study, the frequency characteristics of the SC of the two genetic models are identical.

The post‐SE model of mesial temporal lobe epilepsy has a precipitating brain insult that triggers epileptogenesis, and differs in behavioral, electroencephalographic and neuropathologic features from the three other models.^5^, ^7^, ^42^ Mechanical forces induce injury in the PTE model and this may lead to transient neuroinflammation, axonal injury, and reduced cerebral blood flow.^11^, ^27^, ^28^, ^30^, ^43^, ^44^ These seizures originate as partial seizures from the cortex at the site of injury, and they remain focal or become secondary generalized.^4^, ^7^, ^27^, ^42^ In all, despite all differences in the epileptic phenotype and underlying neural pathways and circuits, as well as that the spike generating site could be near or at distance from the recording electrodes, the AS of the spikes as recorded in the cortex in the four epilepsy models does not differ.

The slow component of the SWC is longer than the SC and occupies the entire period between successive SWCs. The shapes of the slow components were mostly similar in all models, except some post‐SE and PTE animals that had a large late wave in the SWC. However, there was noticeable cross‐model diversity in the frequency of the slow component, which was, on average, higher in the WAG/Rij and GAERS, 8.5 and 8 Hz respectively, and lower in post‐SE and PTE, 4.5 Hz in both models. Notably, the frequency of the individual SWC's slow component is close to the main frequency of seizures/SWDs (reciprocal of the inter‐spike interval). Of note, the frequency properties of the slow components were estimated based on the same number of animals and also on automatically extracted SWCs, but on a somewhat smaller number due to different template patterns. This may have contributed to a larger variability of AS shape and peak frequency across animals of the different models. The larger variability could also be understood in case the shape of the wave is more influenced by local tissue properties and recording characteristics than the shape of the peak.

The similarity of the SC and the differences in the slow components of the SWCs between the animal models allows us to hypothesize that at least two systems participate in the generation of SWCs. One is common and similar across all cortical locations, animals, and epilepsy models/types, while the other shows certain differences that might result from diverse epileptogenic and ictogenesis processes between the models. The difference between models is mostly observed in the second deflection of the biphasic slow component: in WAG/Rij and GAERS, it is short, while in post‐SE, and especially in PTE, it is longer. Other studies have also suggested that the spreading of the spikes and the waves of the SWDs may be governed by distinct mechanisms and circuits.^21^, ^45^, ^46^, ^47^ The latter authors proposed that the spike component is a result of thalamic activation of cortical microcircuits in the subgranular layers of the focal area in the somatosensory cortex, the waves are putatively generated by an intracortical oscillator.^21^

For processing a large number of SCs, we used a proprietary algorithm of pattern recognition. In principle, any spike detection method could be applied as far as it is capable of extracting the SC in its entire duration. A variety of spike detection algorithms is described in the literature.^48^, ^49^, ^50^ However, the main goal of these algorithms is the detection of the spike itself and, as a rule, the time of the spike's peak. An important advantage of our method, which may be classified as a template matching method,^50^ is the possibility to determine the times of onset and offset of the entire SC, which is necessary for proper extraction of the SC and subsequent analysis.

Various spectral analysis methods such as Fast Fourier Transform, wavelet transform,^22^, ^23^, ^51^, ^52^, ^53^, ^54^, ^55^, ^56^, ^57^ or autoregressive analysis^58^, ^59^ have been used primarily to describe the main, or dominant, frequency of the seizure/SWD (i.e., the rate of repetition of SWCs), and its dynamics during the seizure. All these methods require sufficiently long (>1 s) EEG epochs. The present used SBF algorithm may calculate the Fourier transform of signal segments of arbitrary length and allows therefore the analyses of much shorter transient EEG characteristics, such as SWC. Our findings of the universality of SCs in different epilepsy models are that it facilitates the development of reliable seizure detection algorithms. Its suitability to detect and characterize seizures is now experimentally proven on a large number of EEG recordings from different rodent epilepsy models.^6^, ^60^ The results of the current study allowed for improvements in our earlier seizure detection software, particularly in the part of preprocessing; further improvements are possible using the fragmentary decomposition and pattern recognition approach. In this study, we have analyzed the SWCs once the seizures were generalized. Further analysis of SWC properties at the early stages of focal onset seizures (as in post‐SE and PTE models) may help in understanding of mechanisms of epileptogenesis and seizure development.

There is a concern that high‐pass filtering used during EEG acquisition could, in the presence of DC shifts, introduce artificial SWC‐like events in the filtered signal that could affect our results.^38^, ^61^ Although our tests on simulated SWDs with DC shifts showed that the events produced by high‐pass filtering of physiological (slow) DC shifts have significantly longer duration than regular SWCs and thus could not be picked up by our SWC detection algorithm (data not shown), this concern should be considered in further studies where not filtered DC recordings should be used.

## CONFLICT OF INTEREST STATEMENT

None of the authors has any conflict of interest to disclose. We confirm that we have read the Journal’s position on issues involved in ethical publication and affirm that this report is consistent with those guidelines.

## Supporting information


Appendix S1.

Appendix S2.

Appendix S3.

Appendix S4.

Appendix S5.

Appendix S6.

## Data Availability

The data that support the findings of this study are available from the corresponding author upon reasonable request.
