# Supplementary material for: A spike is a spike: On the universality of spike features in four epilepsy models
Source: Epilepsia Open. 2024 Oct 9;9(6):2365–77. doi: 10.1002/epi4.13062 (PMC11633703; doi:10.1002/epi4.13062)
Supplement: Supplementary file 1 — Appendix S1. [file EPI4-9-2365-s002.docx]

# **Supporting Information 1 – Methods**

## Kainic acid induced post-status epilepticus of acquired epilepsy

A repeated low dose kainic acid (KA) administration protocol was used as previously described(Casillas-Espinosa, Sargsyan, et al., 2019; Casillas-Espinosa, Shultz, et al., 2019). Rats were injected i.p. with an initial dose of KA 7.5 mg/kg and monitored for behavioral seizures based on Racine’s scale(Racine, 1972b, 1972a). If no self-sustained seizure activity was observed with at least five class IV-V seizures, another i.p. dose of 2.5 mg/kg of KA was administered up to a maximum of 15 mg/kg. An animal was eliminated from the experiment if it didn’t show a self-sustained SE after the maximum KA dose. SE was stopped after 4 hours with diazepam (5 mg/kg).

## Induction of post-traumatic epilepsy

Wistar rats received a lateral fluid percussion injury (FPI) as previously described(Casillas-Espinosa, Andrade, et al., 2019; Liu et al., 2016; Ndode-Ekane et al., 2019). Briefly, with the animal under anesthesia, a 5-mm craniotomy positioned 2.5-mm left lateral and 5-mm posterior to Bregma, was performed to create a circular window exposing the intact dura mater of the brain. A modified female Luer-Lock cap was secured over the craniotomy window by dental acrylic. A fluid pulse (320–350 kPa) of generated by the fluid percussion device was delivered to the brain. On resumption of spontaneous breathing, and return to pre- FPI levels of heart rate and oxygenation status, the dental acrylic caps were removed and the wound sutured closed. This injury results in PTE in around 30% of rats at 6 months(Brady et al., 2019; Casillas-Espinosa, Andrade, et al., 2019; Kharatishvili et al., 2006; Liu et al., 2016; Shultz et al., 2013).

## ECoG electrode implantation and EEG acquisition

ECoG surgery was performed in all labs under isoflurane anesthesia 10 weeks after SE in the post-SE model, 6 months post- fluid percussion injury in the PTE model, at 6 months of age for the GAERS and between 6 and 8 months for the WAG/Rij. All procedures were previously described(Casillas-Espinosa, Andrade, et al., 2019). In brief, six burr holes were drilled through the skull without penetrating the dura, one on each side of the frontoparietal region (AP: ±1.7; ML: -2.5), two to each side of the temporal region (AP: ±5. 6; ML: left 2.5) anterior to lambda. Stainless steel screw electrodes were screwed into the burr holes, ground and reference electrodes were implanted on each side of the parietal bone above the cerebellum. This setup was used for the GAERS, and the two acquired models. The electrodes were fixed in position by applying Vertex dental cement (Vertex, Australia) around the electrodes and over the skull. WAG/Rij rats received a standard unilateral epidural EEG electrode implantation (Tripolar electrode set from Plastic One, MS 232/2A), with an active electrode above the frontal cortex (AP: 2.0; ML: 2.0, a second one above the parietal cortex (AP: -6.0; ML: -4.0), and a reference electrode above the cerebellum (van Luijtelaar & van Oijen, 2020).

The EEG sampling rate in most of the recordings was 512 Hz. Some recordings of WAG/Rij rats were made with 274.46 Hz sampling rate.

## EEG Analysis

The overall analysis included the following procedures (Figure 1A in main text):

1. Pre-processing: Separation of the original EEG into fast and slow components;
2. Automatic extraction of the SW complexes, done separately for fast and slow components;
3. Calculation of amplitude spectra (AS) of fast and slow components;
4. Statistical analysis of the peak frequencies.

1. Pre-processing

The EEG pre-processing was done in order to separate the original EEG (Figure 1A, trace 1) into low frequency component, which included the baseline trend and the slow waves present in seizures (Figure 1A, trace 2), and the fast component, which included the spikes, or spike complexes (Figure 1A, trace 3). The low frequency component was first calculated using a multiple-pass moving average and then subtracted from the original EEG. The remaining signal is referred to as pre-processed or detrended signal. The detrended signal contains the fast component of the SWC, and the low frequency component contains the slow component of the SWC.

The multiple-pass moving average(Smith S.W., 1999) is a type of finite response filter that performs a simple low-pass filtering. It replaces the current sample by an average of neighboring samples, and this procedure can be repeated several times (multiple passes). A central moving average was used in our calculations, where the mean is taken from an equal number of samples on either side of the central value (this ensures that variations in the mean are aligned with the variations in the original signal rather than being shifted in time). The multiple-pass moving average is defined by two parameters: the moving window size and the number of passes. A window size of 40 ms (20 ms on each side of the current sample) and two passes were used in our calculations, which provided (after the subtraction of the low frequency component from the original EEG) sufficient reduction of the slow waves (Figure 2B, Figure 1A, trace 2). Further, we refer to the pre-processing as detrending rather than filtering to differentiate it from conventional frequency filtering.

2. Automatic extraction of SWCs and their statistical analysis.

To obtain the estimates for the average peak frequencies of fast and slow components of large number of individual SWCs, as well as to calculate the averaged SC's and slow component's time-courses and ASs, a procedure for automated extraction of individual SCs and their slow components from the entire EEG recording of an animal was designed and implemented.

The procedure is based on the proprietary algorithm of pattern recognition in signal model (PRISM). This algorithm consists of two major parts: 1) creating a model of the input EEG by applying the fragmentary decomposition algorithm; 2) model-based detection of specific patterns.

Detailed description of the fragmentary decomposition algorithm can be found in Melkonian et al., 2003(Melkonian et al., 2003). It converts the input signal into a sequence of quasi-Gaussian kernels (QGK) fitted to the half-waves of the input signal. Each QGK is fully defined by three parameters: amplitude, shape and onset time. The sum of these QGKs forms the model signal that accurately resembles the input signal (Figure 1A, trace 4). This transformation of the input signal into the model representation significantly facilitates further processing such as search for specific temporal patterns. In our case, the input signal for model EEG construction was the detrended EEG.

The second part of the PRISM algorithm analyses the model EEG and finds the fragments of model signal (the sequences of QGKs) that match a specific template pattern. The template pattern is also a sequence of QGKs that describes the typical SWC observed in the EEG recording being analyzed.

The PRISM algorithm was then applied to the fast component of the EEG to detect the SCs. The preparation of template pattern for SC detection was done manually for each animal/channel before the automatic processing in the following way: having in front the fragmentary decomposition of the detrended ictal EEG, the user manually selected those QGKs that must form the template pattern, and provided parameter ranges or weights to each template QGK. Template patterns consisting of three QGKs – those corresponding to the three-phasic spike components PT_early_, Sp_2_, and PTlate (Sitnikova & van Luijtelaar, 2007) were used for the detection of SCs.

After the template pattern was defined, the signal processing software run automatically through the entire recording and detected the fragments of the EEG that match the template, i.e., the SCs.

For each detected SC complex, the following computational steps were performed:

1. The start and end time of the SC were defined as shown in Figure 1B and described in figure legend, and the corresponding fragment was extracted from the detrended EEG (Figure 1A, trace 5);
2. The corresponding slow component was extracted from the low frequency component of the EEG calculated at the pre-processing stage (Figure 1A, trace 6). Since the slow component is longer than the SC, its end time was defined by extending the end time defined for the SC by some fixed time interval. This added time interval was defined for each recording before the automatic processing so that it approximately covers the inter-spike interval, but does not overlap with the next spike (Figure 1B).
3. The AS of the SC and slow component were calculated as described in the next section, and the maximum of the AS (the peak) was found and the peak’s frequency was stored for subsequent analysis (Figure 1A, 7).
4. For calculation of the average SC time-course, the currently detected SC was summed with the previous ones. Before summing the current SC was aligned with the previous SC sum using maximum correlation (Pearson’s correlation coefficient was used) so that the best fit of the two time-courses was achieved. The same was done for the slow components.

At the end of the detection procedure, the SC sum was divided by the number of detected SCs to obtain the average SC time course. The AS of the averaged SC was then calculated, and its peak frequency was found (see column 7 in Table 1). The same was done for the slow component (Table 2).

3. Calculation of amplitude spectrum

The AS of the SC and slow component were calculated, and the maximum of the AS (the peak) was found (Figure S1, 7) and the peak’s frequency was stored for subsequent analysis. The amplitude spectrum is defined as

$A(f)=\left| S(f) \right|=\sqrt{(S_{c}(f))^{2}+(S_{s}(f))^{2}}$,

where

$$S(f)=S_{c}(f)-iS_{s}(f)$$

is the complex spectrum, or Fourier transform, of the EEG signal *s*(*t*), $i=\sqrt{-1}$, $S_{c}(f)$ and $S_{s}(f)$ are the real and imaginary parts of the complex spectrum (the cosine and sine Fourier transforms), respectively, and *f* is frequency.

Since the EEG signal for spectral analysis, i.e., the SWC fragment, is defined over a finite time interval, the general procedure of the time to frequency transformation may be presented by the following form of finite Fourier transform

$S(f)=\int_{0}^{T} s(t)\exp(-i2\pi ft)dt$,

where *T* is the duration of the SWC fragment.

The Fourier transform *S(f)* and the amplitude spectrum *A(f)* of the extracted SW complexes were calculated using our previously described similar basis function (SBF) algorithm for Fourier Transform calculation(Melkonian, 2010). In this study a slightly modified version was used, optimized for the uniformly sampled input signals and also faster. The SBF algorithm estimates the sine and cosine Fourier integrals using the function decomposition into a sum of similar basis functions. In contrast to the conventional Fast Fourier transform algorithm (FFT), the SBF algorithm is applicable to short time windows. It may calculate the Fourier transform of signal segments of arbitrary length (including very short ones), with consideration of frequency characteristics as continuous functions of frequency. This provides the possibility to obtain precise frequency estimates of very short EEG epochs. The SBF algorithm is stable in the sense that the increase of the interpolation accuracy makes the numerical solution to converge towards the theoretical one. Conceptually, the SBF algorithm deals with a continuous Fourier spectrum instead of a discrete spectrum defined by the discrete Fourier transform and implemented by the FFT algorithm. The transcription of the continuous functions into a digital form accepts non-uniform sampling intervals in both primary and transformation domains. In the SBF algorithm, the need for windows for spectral analysis is eliminated, along with their distorting impact. Zero-padding is also not necessary.

In this study, the frequency characteristics of the extracted EEG fragments were calculated for the frequency range from 0 Hz to the Nyquist frequency of the corresponding recording (one-half of the sampling rate), and for fixed number of 1000 frequency samples equally distributed in this range.

**References**

Brady, R. D., Casillas-Espinosa, P. M., Agoston, D. v., Bertram, E. H., Kamnaksh, A., Semple, B. D., & Shultz, S. R. (2019). Modelling traumatic brain injury and posttraumatic epilepsy in rodents. *Neurobiology of Disease*, *123*, 8–19. https://doi.org/10.1016/j.nbd.2018.08.007

Casillas-Espinosa, P. M., Andrade, P., Santana-Gomez, C., Paananen, T., Smith, G., Ali, I., Ciszek, R., Ndode-Ekane, X. E., Brady, R. D., Tohka, J., Hudson, M. R., Perucca, P., Braine, E. L., Immonen, R., Puhakka, N., Shultz, S. R., Jones, N. C., Staba, R. J., Pitkänen, A., & O’Brien, T. J. (2019). Harmonization of the pipeline for seizure detection to phenotype post-traumatic epilepsy in a preclinical multicenter study on post-traumatic epileptogenesis. *Epilepsy Research*, *156*, 106131. https://doi.org/10.1016/j.eplepsyres.2019.04.011

Casillas-Espinosa, P. M., Sargsyan, A., Melkonian, D., & O’Brien, T. J. (2019). A universal automated tool for reliable detection of seizures in rodent models of acquired and genetic epilepsy. *Epilepsia*, *60*(4). https://doi.org/10.1111/epi.14691

Casillas-Espinosa, P. M., Shultz, S. R., Braine, E. L., Jones, N. C., Snutch, T. P., Powell, K. L., & O’Brien, T. J. (2019). Disease-modifying effects of a novel T-type calcium channel antagonist, Z944, in a model of temporal lobe epilepsy. *Progress in Neurobiology*, *182*, 101677. https://doi.org/10.1016/j.pneurobio.2019.101677

Kharatishvili, I., Nissinen, J. P., McIntosh, T. K., & Pitkänen, A. (2006). A model of posttraumatic epilepsy induced by lateral fluid-percussion brain injury in rats. *Neuroscience*, *140*(2), 685–697. https://doi.org/10.1016/j.neuroscience.2006.03.012

Liu, S., Zheng, P., Wright, D. K., Dezsi, G., Braine, E., Nguyen, T., Corcoran, N. M., Johnston, L. A., Hovens, C. M., Mayo, J. N., Hudson, M., Shultz, S. R., Jones, N. C., & O’Brien, T. J. (2016). Sodium selenate retards epileptogenesis in acquired epilepsy models reversing changes in protein phosphatase 2A and hyperphosphorylated tau. *Brain*, *139*(7), 1919–1938. https://doi.org/10.1093/brain/aww116

Melkonian, D. (2010). Similar basis function algorithm for numerical estimation of Fourier integrals. *Numerical Algorithms*, *54*(1), 73–100. https://doi.org/10.1007/s11075-009-9324-x

Melkonian, D., Blumenthal, T. D., & Meares, R. (2003). High-resolution fragmentary decomposition—a model-based method of non-stationary electrophysiological signal analysis. *Journal of Neuroscience Methods*, *131*(1–2), 149–159. https://doi.org/10.1016/j.jneumeth.2003.08.005

Ndode-Ekane, X. E., Santana-Gomez, C., Casillas-Espinosa, P. M., Ali, I., Brady, R. D., Smith, G., Andrade, P., Immonen, R., Puhakka, N., Hudson, M. R., Braine, E. L., Shultz, S. R., Staba, R. J., O’Brien, T. J., & Pitkänen, A. (2019). Harmonization of lateral fluid-percussion injury model production and post-injury monitoring in a preclinical multicenter biomarker discovery study on post-traumatic epileptogenesis. *Epilepsy Research*, *151*, 7–16. https://doi.org/10.1016/j.eplepsyres.2019.01.006

Racine, R. J. (1972a). Modification of seizure activity by electrical stimulation: I. after-discharge threshold. *Electroencephalography and Clinical Neurophysiology*, *32*(3), 269–279. https://doi.org/10.1016/0013-4694(72)90176-9

Racine, R. J. (1972b). Modification of seizure activity by electrical stimulation: II. Motor seizure. *Electroencephalography and Clinical Neurophysiology*, *32*(3), 281–294. https://doi.org/10.1016/0013-4694(72)90177-0

Shultz, S. R., Cardamone, L., Liu, Y. R., Hogan, R. E., Maccotta, L., Wright, D. K., Zheng, P., Koe, A., Gregoire, M.-C., Williams, J. P., Hicks, R. J., Jones, N. C., Myers, D. E., O’Brien, T. J., & Bouilleret, V. (2013). Can structural or functional changes following traumatic brain injury in the rat predict epileptic outcome? *Epilepsia*, *54*(7), 1240–1250. https://doi.org/10.1111/epi.12223

Sitnikova, E., & van Luijtelaar, G. (2007). Electroencephalographic characterization of spike-wave discharges in cortex and thalamus in WAG/Rij rats. *Epilepsia*, *48*(12), 2296–2311.

Smith S.W. (1999). *The Scientist and Engineer’s Guide to Digital Signal Processing* (second edition). California Technical Publishing.

van Luijtelaar, G., & van Oijen, G. (2020). Establishing Drug Effects on Electrocorticographic Activity in a Genetic Absence Epilepsy Model: Advances and Pitfalls. *Frontiers in Pharmacology*, *11*. https://doi.org/10.3389/fphar.2020.00395
