# Supplementary material for: A spike is a spike: On the universality of spike features in four epilepsy models
Source: Epilepsia Open. 2024 Oct 9;9(6):2365–77. doi: 10.1002/epi4.13062 (PMC11633703; doi:10.1002/epi4.13062)
Supplement: Supplementary file 2 — Appendix S2. [file EPI4-9-2365-s004.docx]

**Supporting Information 2 – Figures S1 and S2**

SWC

SWD

20 s

Figure S1. Definition of spike-wave complex (SWC) as one cycle of recurring activity during a seizure. A 20 s fragment of WAG/Rij EEG containing a spike-wave discharge (SWD, left trace), enlarged fragment of which is shown at the right.

Post-SE, Animal 1

Post-SE, Animal 2

PTE, Animal 1

PTE, Animal 2

120 s

5 s

5 s

120 s

5 s

5 s

5 s

120 s

5 s

120 s

5 s

5 s

WAG/Rij, Animal 1

20 s

5 s

WAG/Rij, Animal 2

20 s

5 s

GAERS, Animal 1

20 s

5 s

GAERS, Animal 2

20 s

5 s

5 s

5 s

Figure S2. Examples of seizures recorded from four rat models. Seizures are selected arbitrarily from two different animals of each model. Two (for WAG/Rij and GAERS) or three (for post-SE and PTE) traces for each animal are shown; the upper trace shows the entire seizure (20 s EEG fragments for WAG/Rij and GAERS, 120 s EEG fragments for post-SE and PTE); the lower traces show enlarged 5s fragments from the upper trace. The horizontal bars below the upper traces show the position of the corresponding 5s fragment in the seizure. The two 5s fragments for each post-SE or PTE seizure are selected from the initial and the final parts of the seizure, correspondingly, to show the pattern variations.
