# Supplementary material for: A spike is a spike: On the universality of spike features in four epilepsy models
Source: Epilepsia Open. 2024 Oct 9;9(6):2365–77. doi: 10.1002/epi4.13062 (PMC11633703; doi:10.1002/epi4.13062)
Supplement: Supplementary file 3 — Appendix S3. [file EPI4-9-2365-s003.doc]

**Supporting Information 3 – Figure S3**

**WAG/Rij**

**GAERS**

**Post-SE**

**PTE**

**AS**

**SC**

Time, s

Frequency, Hz

**AS**

**SC**

**AS**

**SC**

**AS**

**SC**

Time, s

Frequency, Hz

Time, s

Frequency, Hz

Time, s

Frequency, Hz

Example 1

Example 2

Example 3

17.8

21.1

20.2

21.1

19.4

19.1

21.1

18.3

22.5

20.5

21.6

19.8

Figure S3. Three examples (1-3) of single spike complexes (SC) extracted from the epileptiform EEG of four epilepsy models, and their amplitude spectra (AS, normalized, logarithmic scale for frequency). The vertical scales are in relative units. The numbers near the peaks of the ASs are the peak frequencies, in Hz. The vertical pink stripes are centered at 20 Hz.
