# Supplementary material for: A spike is a spike: On the universality of spike features in four epilepsy models
Source: Epilepsia Open. 2024 Oct 9;9(6):2365–77. doi: 10.1002/epi4.13062 (PMC11633703; doi:10.1002/epi4.13062)
Supplement: Supplementary file 4 — Appendix S4. [file EPI4-9-2365-s006.docx]

**Supporting Information 4 – Tables S4 and S5**

*Table S4. Statistical analysis of SC frequency.*

| **1** | **2** | **3** |  | **4** | **5** | **6** | **7** |
| --- | --- | --- | --- | --- | --- | --- | --- |
| **Model** | **Rat** | **No. of identified SCs** | **AS peak frequency** | | | | |
|  |  |  |  | **AS peak frequency averaged over ASs of every SC** | | **Mode of the distribution histogram of the peak frequencies of every SC** | **Peak frequency of the AS of averaged SC** |
|  |  |  |  | **Average** | **St. Dev.** |  |  |
| **WAG/Rij** | 1 | 2791 |  | 18.2 | 1.95 | 19.0 | 18.2 |
|  | 2 | 85 |  | 17.3 | 1.21 | 18.0 | 17.4 |
|  | 3 | 4205 |  | 19.3 | 2.63 | 19.0 | 20.2 |
|  | 4 | 11402 |  | 20.4 | 2.54 | 20.0 | 20.1 |
|  | 5 | 993 |  | 22.9 | 1.49 | 24.0 | 22.3 |
|  | 6 | 829 |  | 22.5 | 1.65 | 22.0 | 21.8 |
|  | 7 | 1496 |  | 21.7 | 2.02 | 23.0 | 21.3 |
|  | 8 | 1125 |  | 21.4 | 1.08 | 22.0 | 20.9 |
|  | **Total** | **22926** | **Average** | **20.5** |  | **20.9** | **20.3** |
| **GAERS** | 1 | 2004 |  | 17.9 | 1.91 | 18.0 | 18.4 |
|  | 2 | 813 |  | 19.3 | 2.40 | 20.0 | 18.9 |
|  | 3 | 1097 |  | 18.7 | 1.49 | 20.0 | 19.4 |
|  | 4 | 5312 |  | 19.9 | 1.78 | 21.0 | 20.1 |
|  | 5 | 1653 |  | 20.6 | 2.21 | 21.0 | 21.1 |
|  | 6 | 2285 |  | 18.3 | 2.63 | 20.0 | 18.3 |
|  | 7 | 654 |  | 22.1 | 2.23 | 21.0 | 22.5 |
|  | 8 | 4248 |  | 20.3 | 2.70 | 20.0 | 19.8 |
|  | **Total** | **18066** | **Average** | **19.6** |  | **20.1** | **19.8** |
| **Post-SE** | 1 | 282 |  | 20.7 | 3.31 | 23.0 | 19.9 |
|  | 2 | 14215 |  | 19.3 | 5.17 | 17.0 | 17.6 |
|  | 3 | 232 |  | 19.8 | 2.86 | 18.0 | 19.9 |
|  | 4 | 5398 |  | 20.4 | 3.88 | 20.0 | 19.8 |
|  | 5 | 29 |  | 23.5 | 3.00 | 22.0 | 23.6 |
|  | 6 | 2503 |  | 20.0 | 3.82 | 18.0 | 19.2 |
|  | 7 | 2099 |  | 21.1 | 3.32 | 20.0 | 20.6 |
|  | 8 | 200 |  | 22.1 | 2.23 | 23.0 | 21.3 |
|  | **Total** | **24958** | **Average** | **20.8** |  | **20.1** | **20.2** |
| **PTE** | 1 | 216 |  | 18.5 | 1.94 | 19.0 | 18.6 |
|  | 2 | 52 |  | 19.3 | 1.35 | 19.0 | 19.4 |
|  | 3 | 5471 |  | 17.9 | 3.74 | 19.0 | 17.3 |
|  | 4 | 812 |  | 17.8 | 2.27 | 18.0 | 18.3 |
|  | 5 | 199 |  | 20.0 | 2.76 | 22.0 | 19.8 |
|  | 6 | 2628 |  | 18.0 | 3.06 | 19.0 | 17.8 |
|  | 7 | 3631 |  | 21.1 | 3.70 | 23.0 | 19.4 |
|  | 8 | 26697 |  | 20.3 | 3.00 | 20.0 | 20.1 |
|  | **Total** | **38560** | **Average** | **19.1** |  | **19.9** | **18.8** |
|  | **Total** | **104510** | **Average of Averages** | **20.0** |  | **20.3** | **19.8** |

*Table S5. Statistical analysis of slow component frequency.*

| **1** | **2** | **3** |  | **4** | **5** | **6** | **7** |
| --- | --- | --- | --- | --- | --- | --- | --- |
| **Model** | **Rat** | **No. of identified SCs** | **AS peak frequency** | | | | |
|  |  |  |  | **AS peak frequency averaged over ASs of every slow component** | | **Mode of the distribution histogram of the peak frequencies of every slow component** | **Peak frequency of the AS of averaged slow comp.** |
|  |  |  |  | **Average** | **St. Dev.** |  |  |
| **WAG/Rij** | 1 | 2492 |  | 8.5 | 2.30 | 10.0 | 9.4 |
|  | 2 | 38 |  | 5.4 | 1.34 | 7.0 | 9.4 |
|  | 3 | 3596 |  | 7.0 | 2.36 | 7.0 | 6.8 |
|  | 4 | 8558 |  | 6.7 | 2.17 | 7.0 | 8.5 |
|  | 5 | 162 |  | 5.3 | 2.79 | 5.0 | 9.3 |
|  | 6 | 345 |  | 6.6 | 2.87 | 7.0 | 7.5 |
|  | 7 | 382 |  | 5.6 | 2.48 | 7.0 | 8.9 |
|  | 8 | 1016 |  | 7.0 | 1.76 | 7.0 | 8.2 |
|  | **Total** | **16589** | **Average** | **6.5** |  | **7.1** | **8.5** |
| **GAERS** | 1 | 1673 |  | 7.2 | 2.27 | 7.0 | 8.4 |
|  | 2 | 681 |  | 6.8 | 2.10 | 7.0 | 6.6 |
|  | 3 | 975 |  | 7.5 | 2.36 | 6.0 | 7.9 |
|  | 4 | 3734 |  | 6.5 | 2.44 | 7.0 | 8.5 |
|  | 5 | 1322 |  | 7.4 | 2.25 | 8.0 | 8.0 |
|  | 6 | 1984 |  | 7.7 | 2.09 | 8.0 | 8.7 |
|  | 7 | 532 |  | 7.3 | 1.77 | 8.0 | 7.1 |
|  | 8 | 3120 |  | 7.1 | 2.64 | 7.0 | 8.5 |
|  | **Total** | **14021** | **Average** | **7.2** |  | **7.3** | **8.0** |
| **Post-SE** | 1 | 181 |  | 2.4 | 1.36 | 2.0 | 1.9 |
|  | 2 | 12621 |  | 5.3 | 1.82 | 6.0 | 7.4 |
|  | 3 | 232 |  | 5.2 | 3.26 | 7.0 | 6.3 |
|  | 4 | 3898 |  | 5.9 | 2.15 | 6.0 | 9.3 |
|  | 5 | 29 |  | 2.8 | 1.92 | 3.0 | 0.0 |
|  | 6 | 2503 |  | 4.5 | 2.22 | 4.0 | 0.0 |
|  | 7 | 1424 |  | 4.7 | 2.22 | 5.0 | 6.9 |
|  | 8 | 143 |  | 4.3 | 2.22 | 4.0 | 4.0 |
|  | **Total** | **21031** | **Average** | **4.4** |  | **4.6** | **4.5** |
| **PTE** | 1 | 132 |  | 2.4 | 0.91 | 2.0 | 2.8 |
|  | 2 | 25 |  | 3.5 | 1.81 | 2.0 | 3.4 |
|  | 3 | 3707 |  | 2.6 | 1.68 | 2.0 | 2.7 |
|  | 4 | 494 |  | 3.4 | 2.09 | 3.0 | 3.6 |
|  | 5 | 86 |  | 2.5 | 1.26 | 3.0 | 2.6 |
|  | 6 | 1717 |  | 4.5 | 2.49 | 3.0 | 6.3 |
|  | 7 | 2496 |  | 5.2 | 2.31 | 5.0 | 6.7 |
|  | 8 | 22213 |  | 6.4 | 1.99 | 7.0 | 7.7 |
|  | **Total** | **30870** | **Average** | **3.8** |  | **3.4** | **4.5** |
|  | **Total** | **82511** | **Average of Averages** | **5.5** |  | **5.6** | **6.4** |

Note: The individual SWCs' slow components for which the amplitude spectrum had maximum at 0 Hz are excluded when calculating the columns 4, 5, and 6 (otherwise the average frequency would be shifted towards 0), but not from calculating the averaged slow component and its amplitude spectrum - column 7.
