# Supplementary material for: A spike is a spike: On the universality of spike features in four epilepsy models
Source: Epilepsia Open. 2024 Oct 9;9(6):2365–77. doi: 10.1002/epi4.13062 (PMC11633703; doi:10.1002/epi4.13062)
Supplement: Supplementary file 5 — Appendix S5. [file EPI4-9-2365-s005.docx]

**Supporting Information – Considerations Regarding Gibbs Phenomenon**

The Gibbs phenomenon is the oscillatory behavior of the *Fourier series* of a piecewise continuously differentiable periodic function around a jump discontinuity. It reflects the difficulty inherent in approximating a discontinuous function by a *finite* series of continuous sinusoidal waves. Classical filtering methods, e.g., the windowed linear-phase FIR digital filter realized in the fir1 function of Matlab, comprise direct and inverse Fourier transforms via the Fast Fourier Transform (FFT) algorithm, where the limited number of Fourier series coefficients causes the Gibbs phenomenon at sharp transients^1^.

In our study we do not perform filtering in the frequency domain (i.e., we do not change the frequency characteristics and then calculate the inverse Fourier transform), which excludes the possibility of introducing false events in the signal. Smoothing with moving average and then subtracting the smoothed signal from the original also cannot introduce any distortions in the difference signal that were not present in the original signal.

Moreover, the Similar Basis Function (SBF) algorithm^2^ used in our study for Fourier transform calculation to obtain the frequency characteristics of the selected events (see Supporting Information 1) is based on calculation of Fourier integrals, not series, and thus is significantly less subject to the Gibbs phenomenon. This is illustrated in Figure S6. We took a meander-like function of 0.078 s duration with four jump discontinuities, given in 41 samples (sampling rate 512 Hz, black traces in Figure S6 A, B, C), and calculated its sine (imaginary) and cosine (real) Fourier transforms using the SBF algorithm, for frequency range 0 – 500 Hz and for 500 equidistant frequency samples. We then calculated the inverse Fourier transform of the imaginary part (sine Fourier transform) using the same algorithm but for 10 times larger number of samples (410) in time domain (orange trace in Figure S6, A). For comparison, we did the same (direct and inverse Fourier transforms calculation) using the conventional FFT algorithm (“fft” and “ifft” functions of the Numpy library, Python language). The inverse Fourier transform was calculated again for 410 samples in time domain (blue traces in Figure S6, A). Obviously, the Gibbs phenomenon is much less pronounced when the SBF algorithm is used. It becomes smaller when the frequency range for which the direct Fourier transform is calculated is increased to 1000 Hz (Figure S6, B) and almost disappears when the frequency range is increased to 2000 Hz (Figure S6, C).

We then took arbitrarily one spike complex from each animal model, and applied the same procedure, to demonstrate that direct and inverse Fourier transforms with SBF algorithm do not introduce notable Gibbs phenomenon in the real spike complexes, while the FFT does, at least for sharper events (Figure S7). In this Figure, the frequency characteristics are calculated for the frequency range 0 – 500 Hz, and the number of frequency samples = 500.

**A**

**B**

**C**

Figure S6. Behavior of SBF algorithm at jump discontinuities. A. Black trace: a meander-like function of 0.078 s duration with four jump discontinuities, given in 41 sample (sampling rate 512 Hz). Orange trace: result of direct and inverse Fourier transform calculation with the SBF algorithm. The direct Fourier transform is calculated for frequency range 0 – 500 Hz, for 500 equidistant frequency samples. The inverse Fourier transform is calculated for 410 samples in time domain. Blue trace: result of direct and inverse Fourier transforms calculation using the conventional FFT algorithm (fft and ifft functions of the Numpy library, Python language). The inverse Fourier transform is calculated for 410 samples in time domain. It is evident, that the Gibbs phenomenon is much less pronounced when the SBF algorithm is used. It becomes smaller when the frequency range for which the direct Fourier transform is calculated is increased to 1000 Hz (B) and almost disappears when the frequency range is increased to 2000 Hz (C).

0.00

0.05

0.10

**PTE**

**Post-SE**

**GAERS**

**WAG/Rij**

(a)

(b)

(c)

(d)

Time, s

Figure S7. Black traces (a): fragments of the original EEG containing a spike-wave complex, for four rat models. The sampling rate of the original EEG is 274.46 Hz for WAG/Rij example (the shown fragment contains 25 samples) and 512 Hz for other models (the shown fragment of GAERS contains 40 samples, Post-SE – 37 samples, and PTE – 45 samples). Orange traces (b): the result of direct and inverse Fourier transforms using the SBF algorithm. The sine (imaginary) and cosine (real) Fourier transforms of the original spike-wave complex were calculated for the frequency range 0 – 500 Hz, and number of frequency samples = 500, then the inverse Fourier transform of the imaginary part (sine Fourier transform) was calculated for the same samples as the original EEG. Green traces (c): the result of direct and inverse Fourier transforms using the SBF algorithm, where the inverse Fourier transform is calculated for 10 times greater number of samples than the original (i.e., 250 samples for WAG/Rij example, 400 for GAERS, 370 for Post-SE and 450 for PTE). Blue traces (d): the result of direct and inverse Fourier transforms using the conventional FFT algorithm (fft and ifft functions of the Numpy library, Python language), where the inverse Fourier transform is calculated for 10 times greater number of samples than the original (i.e., 250 samples for WAG/Rij example, 400 for GAERS, 370 for Post-SE and 450 for PTE). Comparison of (a), (c) and (d) shows, that (d) clearly demonstrates the Gibbs phenomenon and significantly deviates from (a), while (c) has much smaller (practically negligible) expression of Gibbs phenomenon and is very close to (a).

References:

1. Bénar CG, Chauvière L, Bartolomei F, Wendling F. Pitfalls of high-pass filtering for detecting epileptic oscillations: A technical note on “false” ripples. Clinical Neurophysiology. 2010 Mar;121(3):301–10.

2. Melkonian D. Similar basis function algorithm for numerical estimation of Fourier integrals. Numer Algorithms. 2010 May 19;54(1):73–100.
