# Supplementary material for: A spike is a spike: On the universality of spike features in four epilepsy models
Source: Epilepsia Open. 2024 Oct 9;9(6):2365–77. doi: 10.1002/epi4.13062 (PMC11633703; doi:10.1002/epi4.13062)
Supplement: Supplementary file 6 — Appendix S6. [file EPI4-9-2365-s001.docx]

# **Supporting Information 6 – Illustration of manual extraction of SWC components (Figure S8)**

In the first stage of our study we have extracted the SWC fast and slow components spikes manually, by graphically marking the start and the end of the fast and slow components of the SWC, as shown in Figure S8.

Fast component - SC

Slow component

Fig. S8. Illustration of the manual extraction of the fast component of the SWC - the spike complex (SC, black trace), and the slow component of the SWC (red trace). The vertical dashed lines show the start and end of the SC (black) and the slow component (red). Gray trace is the original EEG.
